# Evaluation of the photoprotective and antioxidant potential of an avobenzone derivative

**DOI:** 10.3389/fphys.2024.1347414

**Published:** 2024-02-29

**Authors:** Ana Júlia Pasuch Gluzezak, Jean Leandro Dos Santos, Silvya Stuchi Maria-Engler, Lorena Rigo Gaspar

**Affiliations:** ^1^ School of Pharmaceutical Sciences of Ribeirão Preto, University of São Paulo, São Paulo, Brazil; ^2^ School of Pharmaceutical Sciences of UNESP, Araraquara, Brazil; ^3^ Department Clin and Toxicol Anal, University of São Paulo, São Paulo, Brazil

**Keywords:** reconstructed human skin, skin cells, avobenzone, antioxidant, photoprotective

## Abstract

Solar radiation can cause damage to the skin, and the use of sunscreens is one of the main protective measures. However, photounstable ultraviolet (UV) filters can generate photoproducts and reactive oxygen species (ROS). Adding antioxidants, such as resveratrol, to enhance the action of UV filters in sunscreens is an interesting strategy for reducing the damage caused by UV radiation exposure. However, new compounds must have their stability, safety and efficacy guaranteed. Avobenzone, a commonly used UV filter, stands out as a promising candidate for structural modification to enhance its stability. Its molecular hybridization with other UV filters and antioxidants can lead to safer and more effective compounds. In this study, the photoprotective and antioxidant potential of a derivative of avobenzone, hybridized with resveratrol’s molecule, was evaluated using *in vitro* models of cells in monolayer and reconstructed human skin (RHS). Phototoxic potential was assessed using fibroblasts, while the antioxidant activity was measured using the DCFH_2_-DA probe in HaCaT keratinocytes and in-house RHS. The derivative exhibited UV absorption and demonstrated photostability. It did not exhibit any phototoxic nor photoreactivity potential. Additionally, it was able to photo stabilize a combination of photounstable UV filters, avobenzone and octyl methoxycinnamate, and to reduce their phototoxic potential. In terms of antioxidant activity, the derivative successfully protected against UVA-induced ROS production in the HaCaT keratinocytes model, showing statistical equivalence to the antioxidant control, quercetin (10 μg/mL). Furthermore, experiments conducted in the RHS model demonstrated a significant reduction of 30.7% in ROS generation compared to the irradiated control. This study demonstrated that structural modifications of avobenzone can lead to the development of a broad spectrum (absorbing UVB and UVA II radiation, as well as a portion of the UVA I radiation), non-phototoxic, non-photoreactive and photostable derivative for sunscreen and anti-aging formulations. This derivative enhances protection against oxidative stress induced by UV radiation and improves the effectiveness of sun protection. In addition to the monolayer model, the use of a standardized in-house RHS model was highly relevant for evaluating the effects of UV radiation and skin aging. This model closely mimics human physiological conditions and enables the testing of new compounds and the investigation of protective mechanisms against skin damage.

## 1 Introduction

The sun provides fundamental energy for the survival of humans and other living beings. However, excessive exposure to solar radiation causes various damages, such as sunburn, premature aging, and even skin cancer. Regular use of sunscreens is one of the main protective measures (Krutmann et al., 2021). Organic UV filters absorb photons from solar radiation and, as a result, the electron is promoted from the ground state to the singlet excited state of higher energy. This molecule in the singlet excited state may return to its ground state or may reach a triplet excited state of lower energy, which can trigger photochemical reactions that lead to the formation of photoproducts (Shaath, 2010; Bonda et al., 2016).

Among the most used UV filters, avobenzone stands out for its ability to absorb in the UVB (280–320 nm) region and mainly in the UVA II (320–340 nm), being one of the few UV filters approved by the Food and Drug Administration (FDA) for UVA-I protection (340–400 nm) (Shaath, 2010; Balogh et al., 2011; Chaudhuri et al., 2016). Nevertheless, it is important to acknowledge that avobenzone undergoes significant degradation in the presence of radiation. Therefore, the photostabilization of UV filters becomes imperative, achieved through their combination with other UV filters like octocrylene and octyl methoxycinnamate, alongside the incorporation of antioxidants such as ubiquinone and trans-resveratrol (Gaspar and Maia Campos, 2006; Silva Scarpin et al., 2021; Afonso et al., 2014; Freitas et al., 2015).

Considering its great importance as an UV filter, avobenzone has become an excellent candidate for structural modification in an attempt to obtain more stable and compatible derivatives with the other components of the formulation (Santana Reis et al., 2014). In light of this, some studies have focused on the discovery of new UV filters and raw materials capable of increasing avobenzone’s photostability and neutralizing the ROS formed during its photofragmentation (Chaudhuri et al., 2006; Jesus et al., 2022).

Reactive oxygen species (ROS) directly affect cellular components as mutagens, as well as indirectly as messengers and regulators in various aspects of cell biology. The term “ROS” encompasses a wide variety of oxidant molecules with several properties and biological functions, including cell signaling and cell damage (Checa and Josep, 2020; Sies et al., 2022). Exposure to UV radiation increases ROS generation, which in turn depletes the levels of the endogenous antioxidant system (Farris and Valacchi, 2022) and enhances cell damage. These damages involve increased permeability of the lysosomal membrane, reduction in the pH gradient, and other changes that gradually become irreversible. As a result, there is a decreased uptake and binding of the neutral red vital dye, allowing for the distinction between viable and damaged or dead cells (OECD TG 432).

Additionally, it is known that UV filters do not effectively protect against ROS. Therefore, the inclusion of antioxidants in photoprotective formulations can offer new ways to prevent oxidative damage caused by ROS (Afaq, 2011; Matsui, 2016; Silva Scarpin et al., 2021). Several studies demonstrate that polyphenols are highly promising antioxidant agents for the prevention of skin damage, particularly skin cancer (Afaq, 2011; Souto et al., 2019; Sajadimajd et al., 2020). Resveratrol, a natural polyphenol, is capable of absorbing radiation across the entire UVB spectrum and part of the UVA spectrum. In addition to its action on oxidative stress, it also exhibits immunomodulatory properties and reduces the levels of inflammation (Nichols and Katiyar, 2010; Afaq, 2011; Hecker et al., 2021; Cui et al., 2022).

Resveratrol acts as an antioxidant compound primarily by neutralizing ROS through the transfer of hydrogen atoms or electrons, followed by the transfer of protons (Constantinescu and Mihis, 2023). Additionally, it can inhibit the oxidation process through enzymatic inhibition (Fauconneau et al., 1997; Acquaviva et al., 2002). Studies demonstrate that resveratrol regulates the levels of cyclooxygenase, lipoxygenase and xanthine oxidase, inhibiting their activities and preventing several diseases (Baur and Sinclair, 2006; Hecker et al., 2021). Furthermore, some analogues of resveratrol have also undergone analysis to prove its photoprotective activity due to their ability to absorb high-energy UV rays and convert them into less energetic molecules (Hudson, et al., 2013). Therefore, the development of new derivatives of UV filters that also possess antioxidant potential is an interesting strategy in the search for new photoprotective compounds. This approach minimizes the cutaneous effects resulting from exposure to UV radiation and simultaneously reduces the damage caused by the formation of ROS (Santana Reis et al., 2014).

**FIGURE 1:**
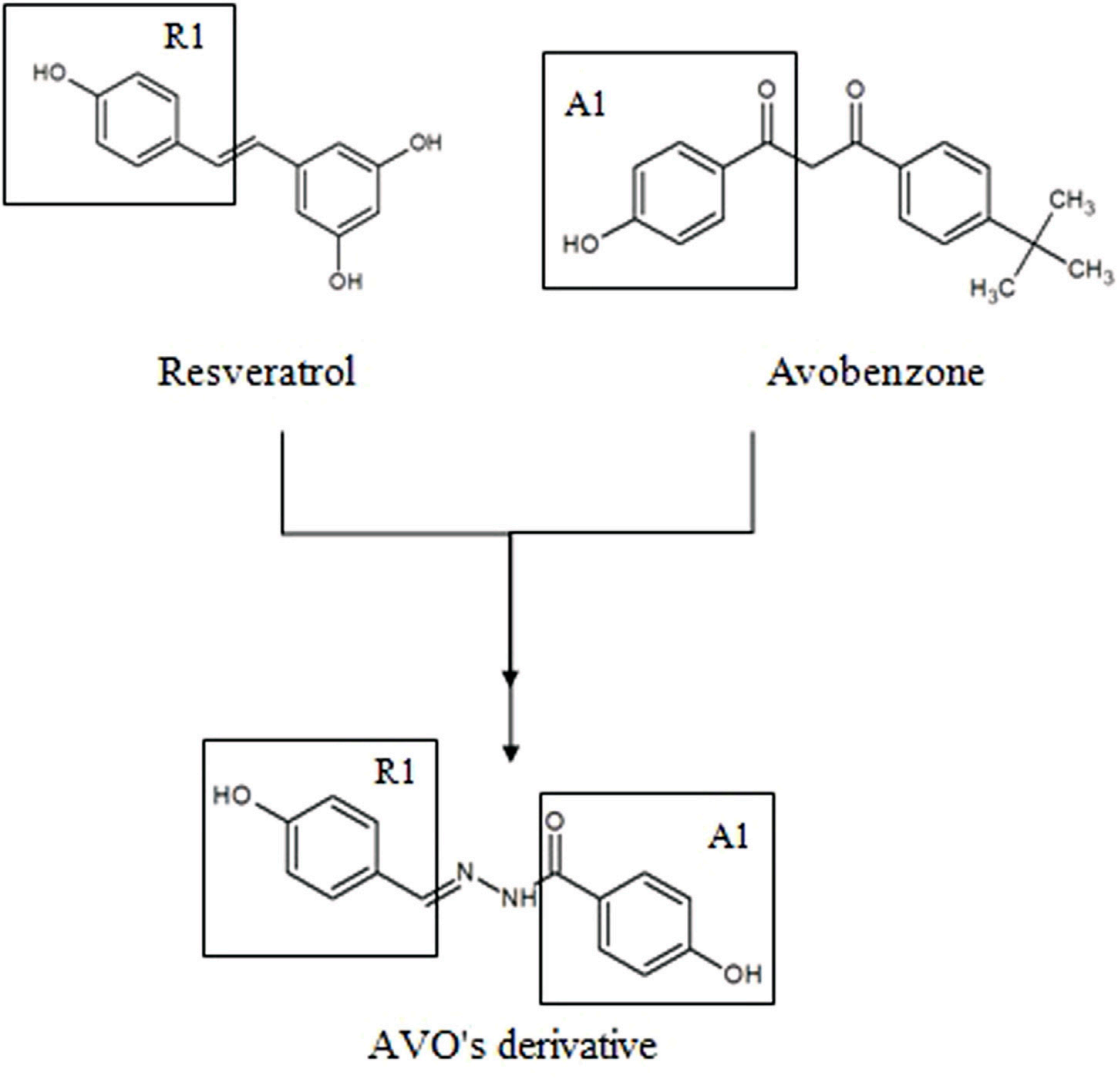
Molecular hybridization among resveratrol and avobenzone subunits to design a new photoprotective compound (AVO’s derivative).

With that in mind, new molecular modifications such as hybridization of avobenzone and resveratrol are proposed in order to obtain more effective, stable and safe compounds. One example is the derivative studied in this study {[(*E*)-4-hidroxi-*N*’-(4-hydroxybenzylidene) benzohydrazide) (LogP: 1.32 ± 0.52)]}, presented in Figure 1. The structure of this derivative comprises a carbonyl connected to a phenol in the *para* position (A1) and a phenol group in the R1 hydroxyl linked to the aromatic group in the *para* position. The N-acylhydrazone subunit serves as the connecting element between both subunits (A1 and R1), facilitating conjugation extension. Each subunit was selected for the synthesis process due to the presence of phenol groups that contribute to the antioxidant activity of this derivative (Santana Reis et al., 2014).

Cell culture systems are crucial tools in various biomedical and clinical studies worldwide, particularly in the development of drugs. The use of 3D reconstructed skin tissues in cell culture systems approximates *in vivo* functionality, providing a cost-effective and efficient alternative to animal testing for preclinical trials. This approach not only reduces the failure rate in drug discovery but also standardizes testing conditions, minimizing experimental variability and project costs. Advances in science and technology are continuously improving these models, increasing their complexity to better reflect the intricate interactions between cells, tissues and organs (de Vries et al., 2015; Amelian et al., 2017; De Vecchi et al., 2018).

Therefore, this study is highly relevant to the cosmetic field because it addresses the evaluation of the photoprotective and antioxidant potential of a derivative of avobenzone, hybridized with a resveratrol molecule, using *in chemico* and *in vitro* models. These models utilize skin cell lines, i.e., fibroblasts and keratinocytes, as well as an in-house standardized reconstructed human skin (RHS) model.

## 2 Materials and methods

### 2.1 Testing substances: UV filters, antioxidant compound and UV filters derivatives

In this study, the UV filters were butyl methoxydibenzoylmethane (avobenzone; Eusolex 9020) from Merck (Darmstadt, DE-HE, Germany) and octyl methoxycinnamate (Uvinul_ MC80) from BASF (Ludwigshafen am Rhein, Germany). The photostabilizer ethylhexyl methoxycrylene (Solastay S1) was obtained from Hallstar Company (Chicago, IL, United States). The antioxidant compound resveratrol was obtained by Fragon from Brazil Pharmaceutical Ltd., and avobenzone derivative [(E)-4-hidroxi-N’-(4-hydroxybenzylidene)benzohydrazide] was obtained by a hybridization process between avobenzone and resveratrol molecules, according to Santana Reis et al., 2014. Ethanol, isopropanol, glycerin and propylene glycol were purchased from Labsynth (Diadema, SP, Brazil). Glacial acetic acid was purchased from J.T. Baker (Phillipsburg, NJ). Alkyl benzoate C12-15 (Crodamol™ AB) and isopropyl myristate (Crodamol ™ IPM) were obtained from Croda (Snaith, United Kingdom). Cetearyl alcohol and cetearyl glucoside (Montanov 68) and hydroxyethyl acrylate/sodium acryloyldimethyl taurate copolymer and squalane and polysorbate 60 (Simulgel_ NS) were obtained from Seppic (Paris, France). Norfloxacin, quercetin, glutamine, antibiotic mixture (penicillin, streptomycin and amphotericin B), dimethyl sulfoxide (DMSO) were purchased from Sigma-Aldrich (St. Louis, MO). The fetal bovine serum and Dulbecco’s modified Eagle medium (DMEM) were purchased from Gibco (Carlsbad, CA). The calf serum was purchased from Hyclone (United States). Neutral red from Merck (Darmstadt, DE-HE, Germany) and cyclomethicone from GE Silicones (Wilton). The radical DPPH (1,1-diphenyl-2-picrylhydrazyl) was purchased from Sigma-Aldrich (Steinheim, Germany). UV filters and avobenzone derivative combinations were tested in a phosphate-buffered saline (PBS) solution and also were added in cosmetic formulations as described in the following items.

### 2.2 Photoprotective potential

#### 2.2.1 Evaluation of photostability by UV spectrophotometry

To assess the photoprotective potential of the new UV filter’s derivative, as well as its combination with avobenzone and octyl methoxycinnamate (Cb), a photostability assay was conducted irradiating all samples at a dose of 27,6 J/cm^2^ emitted by a UVA lamp (Philips UVA Actinic BL/10, Netherlands) (ICH, 1996; 1998), while the negative controls were kept in a dark place. The derivative was tested at 100 μg/mL in isopropanol and the combinations Cb (avobenzone, octyl methoxycinnamate and avobenzone derivative) and Cc (avobenzone, octyl methoxycinnamate and ethylhexyl methoxycrylene were tested with a proportion of 4:8:5 (w/w), where the initial concentrations were 100 μg/mL for octyl methoxycinnamate, 50 μg/mL for avobenzone and 62.5 μg/mL for avobenzone derivative/ethylhexyl methoxycrylene. Solutions were analyzed by spectrophotometry in the 280–400 nm range. The results are expressed as a percentage of the area of irradiated samples related to the area of non-irradiated samples according to Gaspar and Maia Campos (2006) and Tavares et al. (2020).

For the photostability study of the avobenzone derivative in a formulation, experimental formulations were based on a self-emulsifying wax (cetearyl alcohol, cetearyl glucoside) and a liquid polymer and surfactant blend (hydroxyethyl acrylate, sodium acryloyldimethyl taurate copolymer, squalane and polysorbate 60), in accordance to Kawakami and Gaspar (2015) and Silva Scarpin et al. (2021), as described in Table 1. The formulations contained a combination of two UV filters, 8% octyl methoxycinnamate and 4% avobenzone, which are frequently used in sunscreens (Fa). These were supplemented with 0.12% avobenzone derivative (Fb) and 5% of the photostabilizer ethylhexyl methoxycrylene (Fc). There was also a formulation without the UV filters, containing only avobenzone derivative (Fd) to investigate the photoprotective potential.

**TABLE 1:** The formulations and components.

Components	Percentages of components in each formulation (w/w)			
	F_a_	F_b_	F_c_	F_d_
Cetearyl Alcohol (and) Cetearyl Glucoside	3.0	3.0	3.0	3.0
C12-15 Alkyl Benzoate	5.0	5.0	5.0	5.0
Isopropyl Myristate	3.0	3.0	3.0	3.0
Butyl Methoxydibenzoylmethane (avobenzone)	**4.0**	**4.0**	**4.0**	—
Octyl Methoxycinnamate	**8.0**	**8.0**	**8.0**	—
Ethylhexyl Methoxycrylene	—	—	**5.0**	—
BHT	0.05	0.05	0.05	0.05
Glycerin	2.0	2.0	2.0	2.0
Hydroxyethyl Acrylate/Sodium Acryloyldimethyl Taurate Copolymer (and) Squalane (and) Polysorbate 60	1.0	1.0	1.0	1.0
Propylene glycol	2.0	2.0	2.0	2.0
Phenoxyethanol, Methylparaben, Ethylparaben, Propylparaben, Butylparaben	0.8	0.8	0.8	0.8
Cyclomethicone	2.0	2.0	2.0	2.0
(E)-4-hidroxi-N’-(4-hydroxybenzylidene) benzohydrazide) (avobenzone derivative)	—	**0.12**	—	**0.12**
Distilled water	69.15	69.03	64.15	81.15

The bold values in Table 1 mean the presence of the UV filters, and/or UV photostabilizer and/or derivative in the formulations.

According to the BASF Sunscreen Simulator program (BASF. Sunscreen, 2015), the sunscreen formulation containing the UV filters avobenzone and octyl methoxycinnamate was attributed a Sun Protection Factor (SPF) of 15.

The photostability study using a sunscreen was conducted as described in the previous studies of our group, spreading the preparation over an area of 10 cm^2^ (approximately 4 mg/cm^2^) of a glass plate and then left to dry for 15 min. After that, the samples were submitted to an UVA dose of 27.6 J/cm^2^, while others were kept in a dark place (−UV). After irradiation, the plates were immersed in 25 mL of isopropanol and the dried film was dissolved ultrasonically, followed by an analysis using an UV spectrophotometer. The results are expressed as a percentage of the area of irradiated samples related to the area of non-irradiated samples, considered 100% (Gaspar et al., 2013; Kawakami and Gaspar, 2015; Tavares et al., 2020).

#### 2.2.2 Evaluation of photoreactivity

The photoreactivity assay was conducted to detect the generation of oxygen singlet (^1^O_2_; SO) and superoxide anion (O_2_^−•^; SA), according to OECD TG n° 495, as described in Freitas et al. (2015). Avobenzone derivative and the positive (ketoprofen) and negative (L-histidine) controls were tested with final concentrations of 1.0 mM for the avobenzone derivative and 200 mM for the controls, being previously dissolved in DMSO. SO generation was detected by spectrophotometric measurement of p-nitrosodimethylaniline (RNO) bleaching, followed by decreased absorbance of RNO at 440 nm and SA generation was detected by observing the reduction of nitroblue tetrazolium (NBT) at 560 nm. The assay was performed in triplicate in three independent experiments.

According to the ROS Assay protocol (OECD, 2019b), the compound’s photoreactivity is classified according to Table 2.

**TABLE 2:** Criteria for judgment according to values of SO and SA. *Interference: precipitation or color.

Judgment	SO	SA
Photoreactive	≥25	≥70
	<25	≥70
	≥25	<70 and Interference*
Weakly Photoreactive	<25	≥20, <70
Non Photoreactive	<25	<20
Inconclusive	The results do not meet any of the above-mentioned criteria	

### 2.3 Phototoxic assay (3T3 PT NRU)

The 3t3 Neutral red uptake phototoxicity (3t3 NRU PT) test was performed in accordance to OECD TG n° 432 (OECD, 2019a) using 3T3 BALB/c fibroblasts cells from the Cell Bank of Rio de Janeiro (Rio de Janeiro, Brazil). Fibroblasts were cultured in DMEM supplemented with calf serum (10% v/v), L-glutamine (4 mM) and antibiotic mixture (penicillin, streptomycin and amphotericin B) and incubated at 37°C with 5% CO_2_. The DMSO stock solutions of avobenzone derivative was at 100 μg/mL and the combinations Ca (avobenzone and octyl methoxycinnamate) and Cb (avobenzone, octyl methoxycinnamate and avobenzone derivative) were used at proportions of 5:7 and 5:7:5 (w/w), respectively. The initial concentration for the proportion 5:7:5 was 100 μg/mL for octyl methoxycinnamate, 71.4 μg/mL for avobenzone, and 71.4 μg/mL for avobenzone derivative. These preparations were also diluted in PBS (pH 7.2) to generate samples with 8 different concentrations in a geometric progression (constant factor = 1.47). The highest final concentration of DMSO was 1%. Fibroblasts were seeded at a density of 10^4^ cell/well and after 24 h of incubation (5% CO_2_; 37°C), they were treated with 8 different concentrations of the combinations in the wells in sextuplicate, incubated for 1 h (5% CO_2_; 37°C) and irradiated with UVA radiation of 9 J/cm^2^. This dose was selected according to the OECD 432 guideline (OECD, 2019b) for not being cytotoxic and sufficiently potent to excite norfloxacin to elicit phototoxic reactions. For each irradiated plate, there was a negative control (without UVA radiation) that was kept in the dark. After irradiation, the samples tested were replaced with culture medium and the plates were incubated for 18–22 h (5% CO_2_, 37°C).

Cell viability was measured using the NRU assay with the uptake of the vital dye neutral red into cellular lysosomes. Cells were washed with PBS and incubated with culture medium containing 50 μg/mL of the neutral red vital dye for 3 h. Then a solution containing ethanol: water: acetic acid (50:49:1 v/v) was added to the plates. After measuring the absorbance of both plates at 540 nm using a microplate reader (SynergyTM 2, Biotek), all data were analyzed by Phototox Software 2.0 (ZEBET, Germany), and the mean photo effect (MPE) was calculated. According to the prediction model, a test substance with a MPE >0.15 is predicted to be “phototoxic,” a MPE >0.1 and <0.15 is predicted to be “equivocal phototoxic” and a MPE <0.1 is predicted to be “non-phototoxic” (OECD, 2019a).

### 2.4 Antioxidant potential

#### 2.4.1 DPPH free radical scavenging

The DPPH free radical scavenging method was conducted according to Martins et al. (2016), with modifications. This assay is based on the reduction of the 2,2-diphenyl-1-picrylhydrazyl radical by the antioxidant agent, acting as a hydrogen donor. DPPH is a stable radical due to its molecular conformation, capable of reacting with hydrogen/electron donors, generating its reduced form, accompanied by a reduction in its absorbance (517 nm) (Kedare and Singh, 2011). The antioxidant activity is then determined by monitoring the change in the color of the reaction medium, changing from violet to yellow (Pisoschi and NEgulescu, 2012).

In a 96-well plate, 100 µL of each sample were diluted in methanol. The concentrations used for avobenzone derivative under study were 100–0.5375 μg/mL (constant factor: 2) and quercetin was used as a positive control for the test (14–0.109 μg/mL, constant factor: 2). The plate was covered and kept in a dark place for 30 min at room temperature. The absorbance reading was performed at 517 nm using a microplate reader (Synergy™ 2#, Biotek, United States), and the results obtained were used to calculate the inhibition percentages. The assay was performed in triplicate in three independent experiments. The dose-response curve was constructed using the value of inhibition percentage versus concentration; thus, the linear equation and the *R* ^2^ (≥0.95) were obtained. The half maximum inhibitory concentration (IC_50_-DPPH) was calculated using the linear equation by replacing the value of y with 50 (Chen et al., 2013).

#### 2.4.2 Protection against intracellular UVA-induced ROS production

##### 2.4.2.1 In HaCaT keratinocytes monolayer model

Prior to the beginning of the assay, cell viability was evaluated to exclude the possibility that the decrease in fluorescence intensity was related to cell death and not to antioxidant activity. HaCaT keratinocytes were cultured in DMEM supplemented with fetal bovine serum (10%), pyruvate (1 mM), penicillin (100 IU/mL) and streptomycin (100 μg/mL), seeded in 96-well plates (1 × 10^5^ cells/well) and, 24 h later, treated with avobenzone derivative (31,25–1000 μg/mL; constant factor: 2). Cell viability was determined using the NRU assay (Gaspar et al., 2013; Rangel et al., 2020) and sodium dodecyl sulfate (SDS) was used as a positive control (100 μg/mL). Untreated cells absorbance at 540 nm were considered 100% of cell viability to calculate the percentage relative to each sample tested.

To evaluate intracellular ROS production, such as hydroxyl radical, hydrogen peroxide, nitrite and carbonate anion, the probe 2,7-dichlorodihydrofluorescein-diacetate (DCFH_2_-DA) was used. This probe can undergo hydrolysis by cellular esterases forming the compound DCFH, that can be converted by ROS into the fluorescent compound 2,7-dichlorodihydrofluorescein (DCF), measured at the end of the assay (Kalyanaraman et al., 2012; Alam et al., 2013). The assay was performed under the same conditions (cell type, density and exposure times) as the evaluation of cell viability in immortalized HaCaT keratinocytes.

In detail, 1 × 10^5^ cells/well of HaCaT keratinocytes were seeded in 96-well plates and incubated for 24 h at 37°C in a 5% CO_2_ atmosphere. Then, cells were treated with the avobenzone derivative (31,25–1,000 μg/mL; constant factor: 2) for 1 h and the probe DCFH_2_-DA (10 µM) for 30 min, followed by 4 J/cm^2^ of UVA radiation (Solar simulator Dr. Hönle type SOL-500, Planegg, Germany). This UV waveband was chosen because UVA and visible light are the most relevant radiations for ERO production (Zastrow et al., 2009; Vandersee et al., 2015). Quercetin (10 μg/mL) and norfloxacin (100 μg/mL) were used as antioxidant and pro-oxidant controls, respectively. The plates were measured using a microplate reader (Synergy™ HT, BioTek, United States) at 485 nm excitation and 528 nm emission. Results are expressed as a percentage of fluorescence in relation to the irradiated control, considered 100%.

##### 2.4.2.2 In reconstructed human skin model—RHS

In-house RHS models were prepared on 24-well cell culture inserts, with primary human fibroblasts and keratinocytes from foreskin (pooled from three donors) after approval by the Ethics Committee in Research Involving Human Beings—School of Pharmaceutical Sciences of Ribeirão Preto—USP (CAAE n° 35685220.3.0000.5403; Protocol CEP/FCFRP n° 551).

Primarily, the dermal compartment consisted of collagen type I (Corning^®^) and 1.14 × 10^5^ normal human fibroblasts that were seeded into the insert (0.4 µm pore size; ThinCert™, Greiner Bio-One GmbH, Frickenhausen—Germany) and incubated overnight. After 20 h, 3.7 × 10^5^ normal human keratinocytes were seeded on the top of the dermis mixture and kept submerged in an in-house prepared culture medium for 24 h, so the cells could form a monolayer. The models were maintained at an air-liquid interface throughout 7 days, changing the media every 2 days, allowing complete epidermis differentiation and stratification (Pennacchi et al., 2015; PIVETTA et al., 2019; Tavares et al., 2020). Skin samples were fixed in formalin (4%) and submitted to a routine procedure and subsequent staining with hematoxylin-eosin for histological analysis of the morphological development of the models under study.

After the RHS were fully differentiated on day 10, they were placed into new 24-well plates and the measurement of intracellular ROS production began incubating them in the dark with DCFH_2_-DA probe (50 µM) for 45 min. After PBS washing, 25 µL were applied on top of each skin model for 1 h, of avobenzone derivative (200 μg/mL) and the combination Cb (avobenzone, octyl methoxycinnamate and avobenzone derivative) in the proportion of 4:8:5 (w/w), where the initial concentrations were 320 μg/mL for octyl methoxycinnamate, 160 μg/mL for avobenzone and 200 μg/mL for avobenzone derivative. Then, the tissues were submitted (+UV) or not (−UV) to 10 J/cm^2^ of UVA radiation from a solar simulator (SOL-500 Dr Honle AG, Planegg, Germany).

Immediately after irradiation and washing with PBS, the tissues were frozen in liquid nitrogen and 8 µm histological sections were obtained in a cryostat. Pictures were taken in an inverted Ti-S microscope (Nikon Instruments Inc., Holland), 488 nm, using 100 ms of exposure intensity and analyzed by ImageJ software (Rasmussen et al., 2010; Marionnet et al., 2014). Results of fluorescence intensity were normalized to area/pixels and expressed as percent fluorescence compared to untreated irradiated (NT + UV) control, considered 100%.

### 2.5 Statistical analysis

The data were derived from three independent experiments and are reported as mean ± standard error. Significance level was set at *p* ≤ 0.05 for exploratory data analysis. Statistical analysis was conducted using One-way ANOVA followed by Tukey *post hoc* tests.

## 3 Results

### 3.1 Photoprotective potential

**FIGURE 2:**
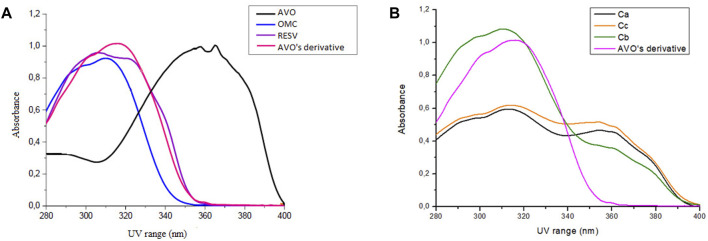
Absorption spectra in the UV region (280–400 nm) of **(A)** The isolated UV filters avobenzone (AVO) and octyl methoxycinnamate (OMC), the antioxidant compound *t*-resveratrol (RESV) and avobenzone derivative (AVO’ derivative) (100 μg/mL); **(B)** The combinations Ca (avobenzone and octyl methoxycinnamate); Cb (avobenzone, octyl methoxycinnamate and avobenzone derivative); Cc (avobenzone, octyl methoxycinnamate and ethylhexyl methoxycrylene). Results are expressed as mean absorbance (*n* = 3).

Avobenzone derivative absorbed mainly in the UVB (280–320 nm) and UVA II (320–340 nm) regions and in a part of the UVA I region (340–360 nm), being quite similar to resveratrol and octyl methoxycinnamate (Figure 2A), while avobenzone mainly absorbs in the UVA I and II regions (320–400 nm), but also absorbs in the UVB (280–320 nm). The combination Ca (avobenzone and octyl methoxycinnamate) (Figure 2B) presented a broad-spectrum UV absorption, and the same was observed by adding the photostabilizer ethylhexyl methoxycrylene (Cc). On the other hand, when avobenzone derivative was added to the combination of UV filters (Cb), a pronounced increase in the UVB absorption was observed (Figure 2B).

**FIGURE 3:**
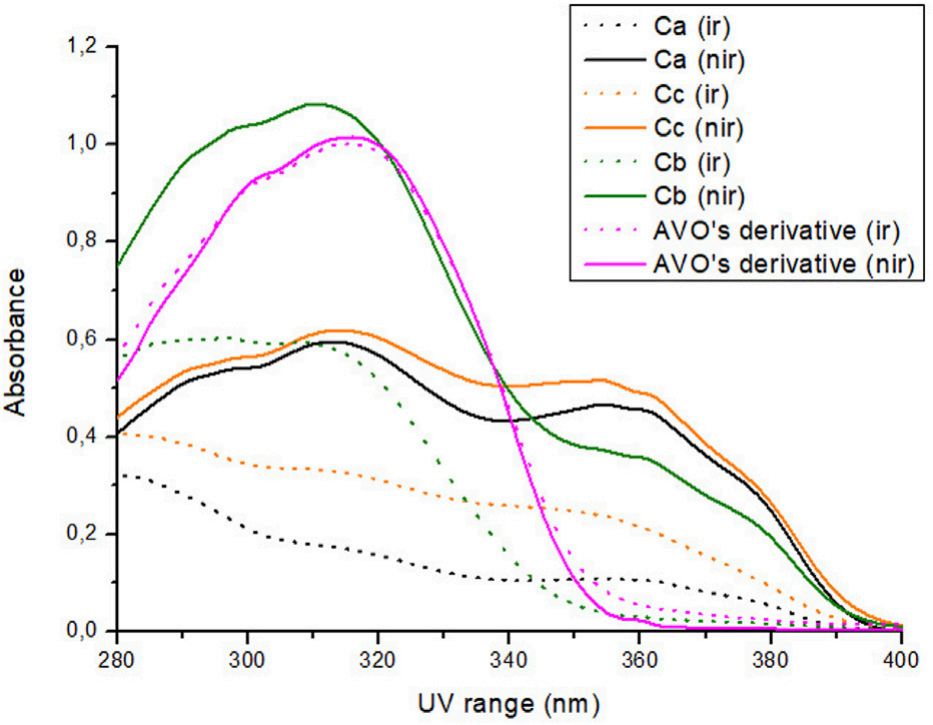
Absorption spectra of the samples in solution before (nir) and after (ir) UVA irradiation. Ca: avobenzone and octyl methoxycinnamate; Cb: avobenzone, octyl methoxycinnamate and avobenzone derivative; Cc: avobenzone, octyl methoxycinnamate and ethylhexyl methoxycrylene. Results are expressed as mean absorbance (*n* = 3).

Regarding their photostability, when irradiated at 26.7 J/cm^2^ of UVA radiation, according to Figure 3; Table 3, UV filters avobenzone and octyl methoxycinnamate were considered photounstable (both the solution Ca and formulation Fa), with statistical difference between the irradiated and non-irradiated pair (*p* < 0.05). When the photostabilizer ethylhexyl methoxycrylene was added to the solution of UV filters (combination Cc), it was still considered photounstable (*p* < 0.05), but it was able to increase UV absorption, with remaining values that went from 23.7 (Ca) to 43.2 (Cc) in the UVA spectrum, and from 42.8 (Ca) to 63.6 (Cc) in the UVB spectrum (*p* < 0.05). The solution of UV filters and avobenzone derivative (combination Cb) was considered photounstable (*p* < 0.05), but, as shown in Figure 3, the addition of the derivative to the UV filters solution was able to increase the absorption in the UVB spectrum, as well as reduce the photodegradation in this region, with remaining values changing from 42.8 (Ca) to 58.7 (Cb) (*p* < 0.05).

**TABLE 3:** Mean of remaining absorption values of the area under the curve of the irradiated solutions and formulations (+UV) compared to non-irradiated ones (−UV) considered 100% in the UV range.

Sample		Remaining absorption values after irradiation (%)		Stability classification
		UVA	UVB	
Solutions	Ca	23.7 ± 3.2	42.8 ± 3.2	Photounstable*
	Cb	28.9 ± 1.2	58.7 ± 0.7^a^	Photounstable*
	Cc	46.2 ± 0.8^a^	63.6 ± 2.3^a^	Photounstable*
	avobenzone derivative	97.7 ± 2.5	97.8 ± 2.1	Photostable
Formulations	Fa	81.8 ± 0.9	91.5 ± 0.2	Photounstable*
	Fb	91.8° ± 1.8°	96.3° ± 0.9°	Photostable
	Fc	98.2 ± 2.2	95.4 ± 4.9	Photostable
	Fd	117.9° ± 0.8°	115.1° ± 2.3°	Photostable

Solutions Ca: avobenzone and octyl methoxycinnamate; Cb: avobenzone, octyl methoxycinnamate and avobenzone derivative; Cc: avobenzone, octyl methoxycinnamate and ethylhexyl methoxycrylene; formulations Fa: avobenzone and octyl methoxycinnamate; Fb: avobenzone, octyl methoxycinnamate and avobenzone derivative; Fc: avobenzone, octyl methoxycinnamate and ethylhexyl methoxycrylene; Fd: formulation with only avobenzone derivative. *: Statistically different from its non-irradiated pair (*p* < 0.05); ^a^: Statistically different from Ca; °: Statistically different from Fa (*p* < 0.05).

For the avobenzone derivative, both the solution and the formulation Fd were considered photostable, since the irradiated pairs were statistically equal to their non-irradiated pairs (*p* > 0.05). When added to the photounstable formulation with the UV filters (Fb), it was able to act as a photostabilizer, increasing the remaining UV absorption values when compared to Fa (*p* < 0.05), resembling the commercial photostabilizer ethylhexyl methoxycrylene effect in Fc (Table 3).

The results for the photoreactivity assay show that the positive control (ketoprofen) was classified as photoreactive with values of SO and SA according to OECD TG 495 (SO: 120–346; SA: 77–151), and the negative control (L-histidine) was classified as non-photoreactive, with values within the recommended range (SO: −8 a 12; SA: 8 a 120). Avobenzone derivative was also considered non-photoreactive, with SO values under 25 and SA values under 20 (Table 4).

**TABLE 4:** Photoreactivity values for SO and SA according to OECD TG 495. Results are expressed as mean ± standard errors of the mean (*n* = 3 independent experiments).

Sample	SO	SA	Classification	
Ketoprofen	283.6 ± 30.4	116 ± 20.6	120–346 (SO)	Photoreactive
			77–151 (SA)	
L-histidine	0.56 ± 13.4	29.8 ± 10.6	−8–12 (SO)	Non-photoreactive
			8–120 (SA)	
Avobenzone derivative	−24.3 ± 22.7	0.35 ± 28.4	<25 (SO) and <20 (SA)	Non-photoreactive

**FIGURE 4:**
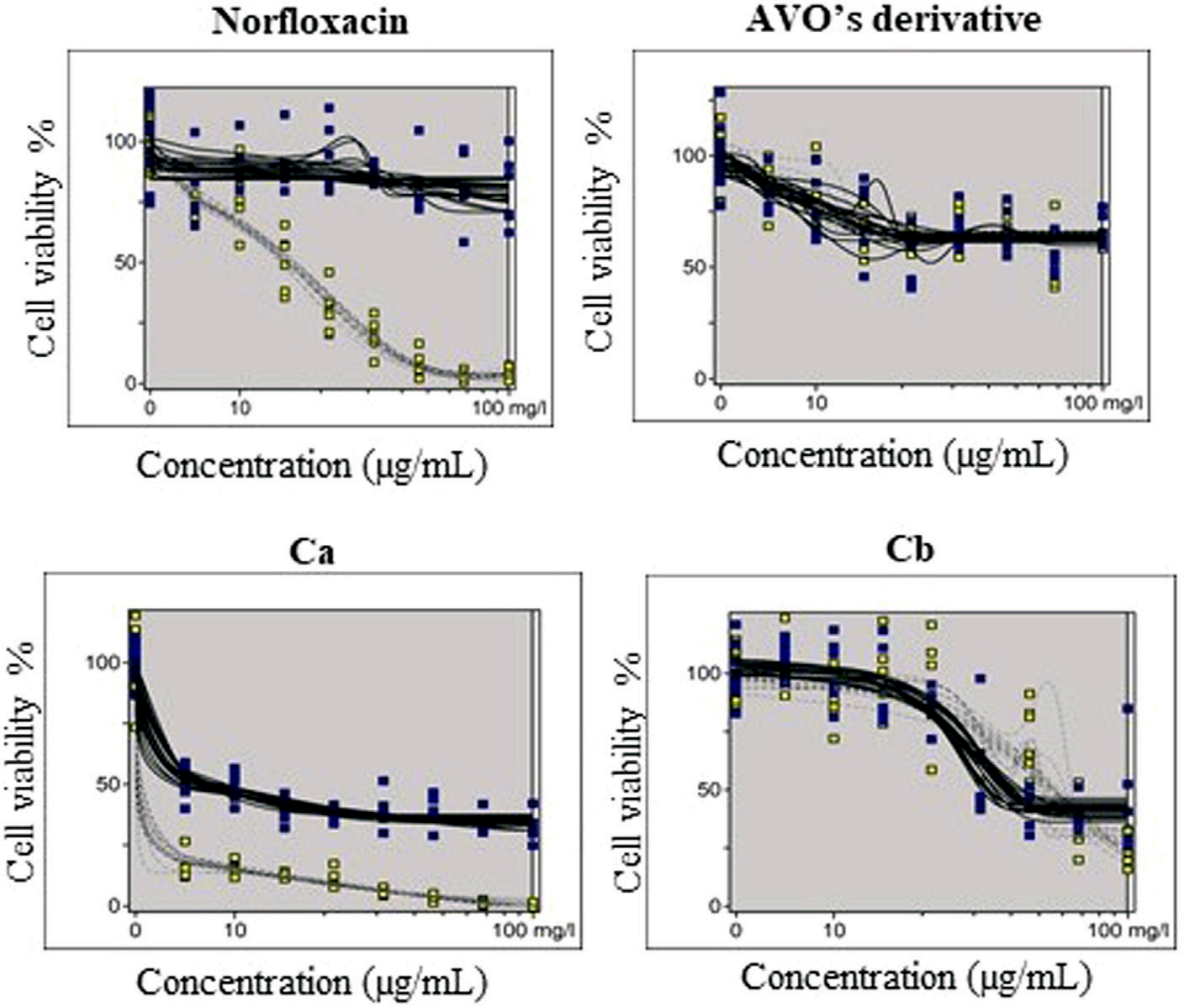
Dose-response curves of avobenzone derivative and the combinations Ca (avobenzone and octyl methoxycinnamate) and Cb (avobenzone, octyl methoxycinnamate and avobenzone derivative), and norfloxacin, used as positive control, obtained by the 3T3 NRU phototoxicity test and plotted using the Phototox Version 2.0 software. The blue and yellow dots refer to non-irradiated (−UV) and irradiated (+UV) substances, respectively.

The phototoxic potential results show that Norfloxacin (positive control) was predicted as phototoxic, with MPE values within the recommended OECD TG 432 range (MPE: 0.34–0.9). The combination Ca (avobenzone and octyl methoxycinnamate) in the proportion of 5:7 (w/w) was considered phototoxic (MPE >0.150), and the addition of avobenzone derivative to this combination (Cb) was able to reduce its phototoxicity (MPE <0.100). Avobenzone derivative alone was also considered non phototoxic (MPE <0.100) (Table 5; Figure 4).

**TABLE 5:** Results for 3T3 NRU phototoxicity assay for the samples: norfloxacin, positive control; avobenzone derivative; combination Ca (avobenzone and octyl methoxycinnamate); combination Cb (avobenzone, octyl methoxycinnamate and avobenzone derivative). Results are expressed as mean photo effects (MPE) of two independent experiments.

Sample	IC_50_ -UV	MPE	Prediction model
Norfloxacin	—	0.568	Phototoxic
	—	0.520	
Avobenzone derivative	—	−0.009	Non-Phototoxic
	—	−0.016	
Ca	7.744	0.302	Phototoxic
	36.714	0.233	
Cb	2.624	0.016	Non-Phototoxic
	4.481	0.018	

### 3.2 Antioxidant potential

After the non-phototoxic response that was observed in the prediction model using fibroblasts, the first antioxidant potential method was conducted with the *in chemico* scavenging of the free radical DPPH. The results demonstrated a promising antioxidant potential for avobenzone derivative, which presented an IC_50_ value of 18.2 ± 10.2 μg/mL, while the antioxidant control, quercetin, presented an IC_50_ value of 2.85 ± 1.03 μg/mL.

**FIGURE 5:**
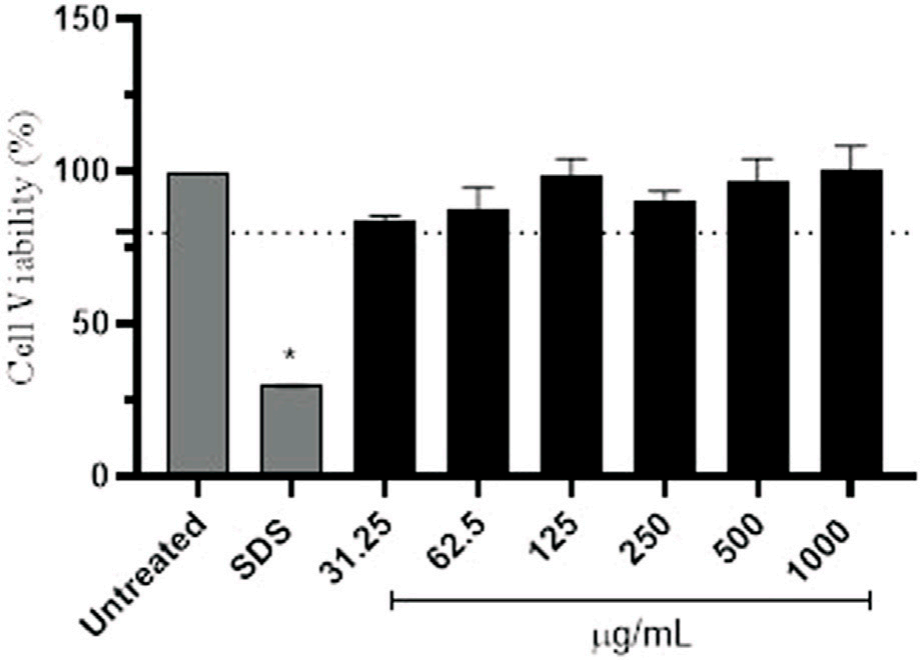
Percentage of cell viability in HaCaT cells after treatment with different concentrations (μg/mL) of the avobenzone derivative. Sodium lauryl sulfate (SDS) was used as positive control. Avobenzone derivative was tested in the range of 31.25–1,000 μg/mL (constant factor: 2). Test substances below the 70% threshold (dotted line) are considered cytotoxic. The results are expressed as mean ± standard errors of the mean of three independent experiments (*n* = 3). *: Significantly different from the untreated non-irradiated control (NT -UV) (*p* < 0.05).

Then, the protective effect of the avobenzone derivative was evaluated by the detection of intracellular ROS immediately after UVA radiation using the probe DCFH_2_-DA, firstly in HaCaT keratinocytes and also in a RHS model. Preliminary experiments for the assessment of cell viability of keratinocytes showed that all tested concentrations of the avobenzone derivative were not cytotoxic for HaCaT cells, with only SDS being statistically cytotoxic compared to the untreated control (*p* < 0.05) and below the 70% threshold, according to the ISO 10993-5, 2009 (Figure 5).

**FIGURE 6:**
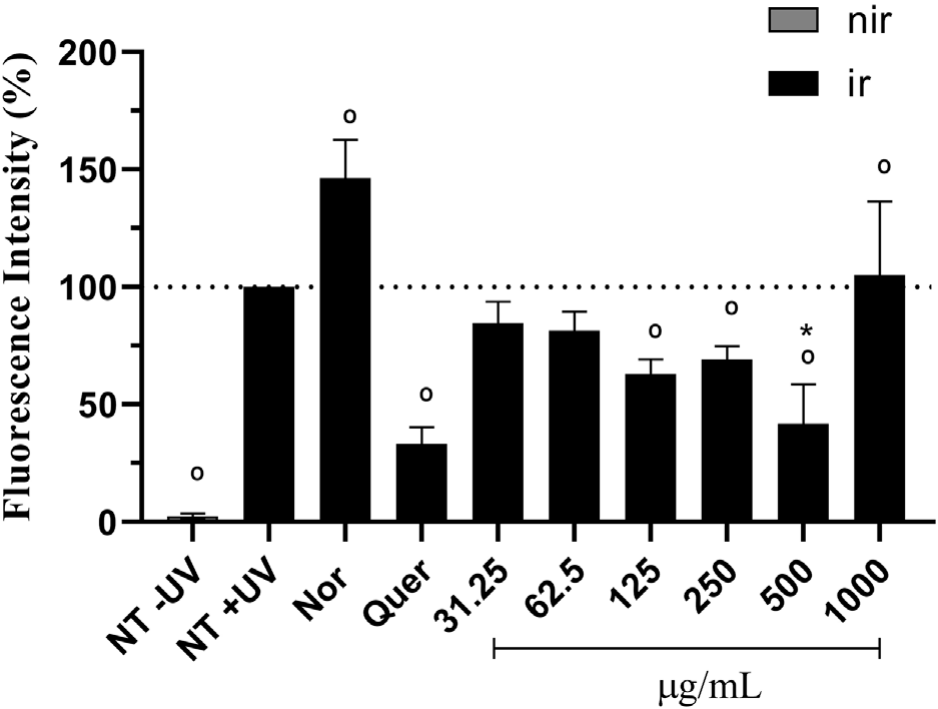
Protection against UVA-induced intracellular ROS production in HaCaT keratinocytes. The results are expressed as a percentage of fluorescence. NT -UV: untreated non-irradiated control; NT + UV: untreated irradiated control; Nor: norfloxacin (100 μg/mL); Quer: quercetin (10 μg/mL); Avobenzone derivative tested in the range of 31.25–1,000 μg/mL (constant factor: 2); Nir: non-irradiated cells; Ir: irradiated cells. Results are expressed as mean ± standard errors of the mean of three independent experiments (*n* = 3). °: Significantly different from the irradiated untreated control (NT + UV) control (*p* < 0.05); *: Statistically equal to quercetin (*p* > 0.05).

UVA irradiation induced ROS generation in keratinocytes (100%) when compared to the untreated non-irradiated control (NT -UV) (2.45%). The pro-oxidant control, norfloxacin (100 μg/mL), significantly increased ROS production by approx. 46% and the antioxidant control, quercetin (10 μg/mL), significantly decreased ROS production by approx. 67%, when compared to the NT + UV (*p* < 0.05) (Figure 6). Avobenzone derivative was able to protect HaCaT cells from ROS generation, demonstrating a significant reduction of 37, 31% and 58% in ROS generation compared to the irradiated control NT + UV (*p* < 0.05), when tested at 125, 250, and 500 μg/mL, respectively. The derivative at 500 μg/mL showed statistical equivalence to the antioxidant control, quercetin (*p* > 0.05). The higher tested concentration, 1,000 μg/mL, showed a possible pro-oxidant effect, since it presented statistically higher values (5%) than NT + UV (*p* < 0.05). The non-irradiated (−UV) tissues treated with quercetin, norfloxacin and avobenzone derivative showed low basal values of fluorescence intensity (ranging from 0.96% to 4.93%), similar to the NT -UV (2.45%).

**FIGURE 7:**
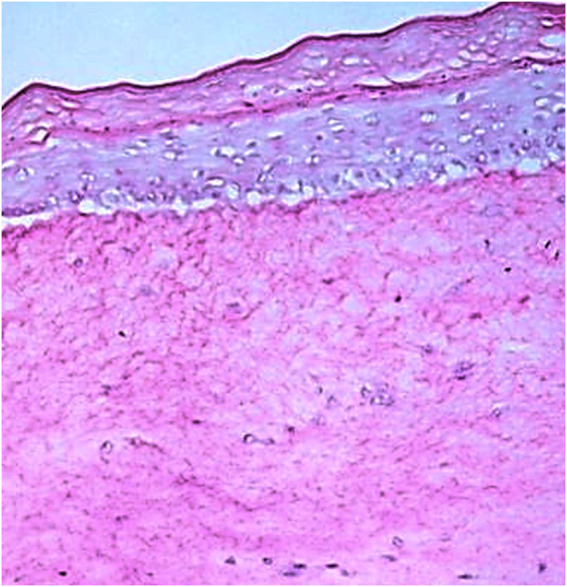
Histologic slide obtained by Hematoxylin-Eosin staining showing all layers of the in-house RHS. Scale bar: 200 μm.

The RHS models after 10 days of culture resulted in a stratified epidermis with well-expressed *stratum basale, spinosum, granulosum,* and *corneum* (Figure 7), that is able to assess the protection against UVA-induced intracellular ROS generation.

**FIGURE 8:**
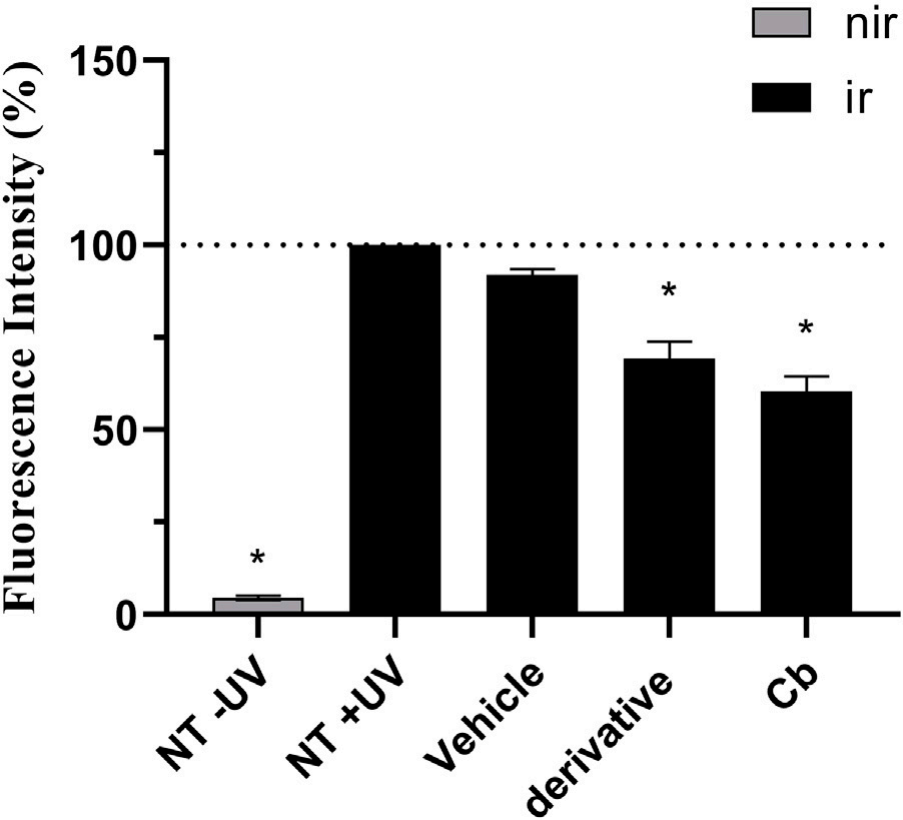
UVA-induced intracellular ROS production in the RHS model. The results are expressed as a percentage of fluorescence in comparison to the NT + UV. Untreated non-irradiated control (NT -UV); Untreated irradiated control (NT + UV); Vehicle, PBS with ethanol 2%; Avobenzone derivative (200 μg/mL); Combination Cb (avobenzone, octyl methoxycinnamate and avobenzone derivative). Nir: non-irradiated tissue; Ir: irradiated tissue. Results are expressed as mean ± standard errors of the mean of three independent experiments (*n* = 3). *: Significantly different from the untreated irradiated control (NT + UV) (*p* < 0.05).

**FIGURE 9:**
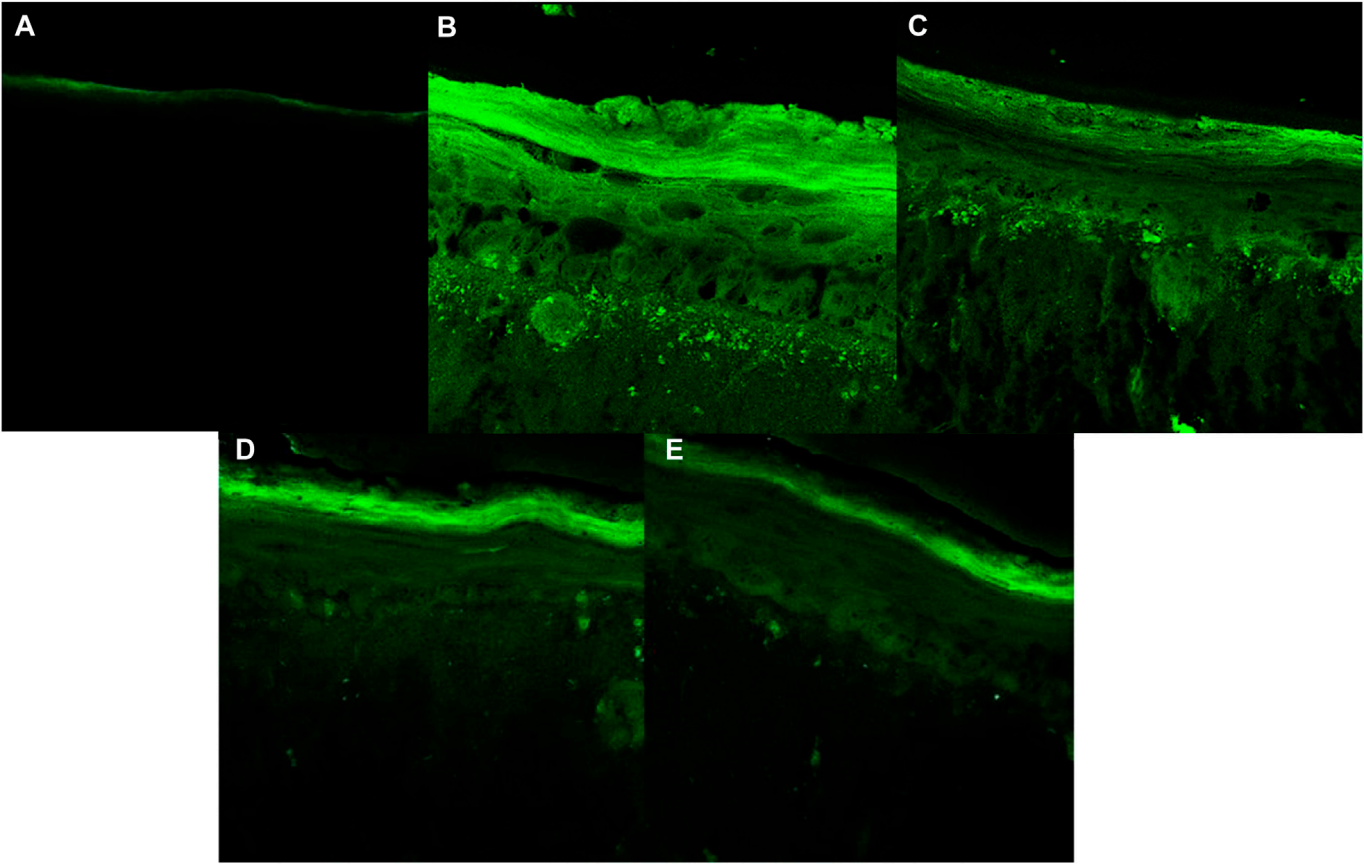
Fluorescence images obtained for confocal microscopy for each treatment: **(A)** Untreated non-irradiated tissue (NT -UV); **(B)** Untreated irradiated tissue (NT + UV); **(C)** Vehicle PBS with ethanol 2%; **(D)** Avobenzone derivative (200 μg/mL); **(E)** Combination Cb (avobenzone, octyl methoxycinnamate and avobenzone derivative) (4:8:5 w/w). Green fluorescence corresponds to the presence of ROS in the RHS.

Figures 8, 9 show that the UVA radiation generated ROS in the untreated irradiated RHS control (NT + UV) (100%), when compared to the untreated non-irradiated control (NT -UV) (*p* < 0.05). The vehicle (PBS with ethanol 2%) reduced only 8% of ROS formation, while avobenzone derivative was able to protect the RHS model, when tested at 200 μg/mL, demonstrating a significant reduction of 30.7% in ROS generation compared to the NT + UV control (*p* < 0.05). When added to the photounstable combination of UV filters, avobenzone and octyl methoxycinnamate, ROS production was reduced by 39.5% (*p* < 0.05), indicating a photo stabilizing effect of the derivative.

## 4 Discussion

Photoprotective formulations must effectively protect the skin against the harmful effects of UV radiation without posing any health risks to consumers. Therefore, achieving broad-spectrum absorption in both the UVA and UVB regions, as well as ensuring the photostability of UV filters, is crucial. Photodegradation reactions can generate ROS, compromising the safety and efficacy of the formulation. Thus, the addition of antioxidants, such as resveratrol, in sunscreens to enhance the action of UV filters is an interesting strategy for reducing the damage caused by UV radiation exposure (Freitas et al.,. 2015). Various molecular modification strategies have been explored to obtain derivatives with broad-spectrum absorption in the UV region and higher antioxidant potential (Santana Reis et al., 2014), leading to safer and more effective compounds. In this study, an avobenzone derivative was evaluated for its photoprotective and antioxidant potential using cells in monolayer and RHS models. The synergistic effects of the derivative in combination with a photounstable combination of UV filters, avobenzone and octyl methoxycinnamate, was also examined.

The UV absorption profile of the avobenzone derivative was investigated as an initial step in characterizing its photoprotective properties. The derivative exhibited an absorption primarily in the UVB (280–320 nm) and UVA II (320–340 nm) regions as well as a portion of the UVA I (340–360 nm) region. This absorption profile is similar to that of resveratrol and octyl methoxycinnamate, while avobenzone itself absorbs in the UVA region (320–400 nm) (Rangel et al., 2020; Silva Scarpin et al., 2021). The absorption spectra for avobenzone derivative can be explained by its chemical structure, since it is formed by aromatic rings, conjugated double bonds, and electron donor and acceptor groups resulting from the hybridization process with avobenzone and resveratrol molecules, as described by Santana Reis et al. (2014). Notably, the addition of the avobenzone derivative to the photounstable combination of UV filters resulted in a significant increase in absorption in the UVB region.

The combination of avobenzone and octyl methoxycinnamate exhibited broad-spectrum UV absorption and was found to be photounstable, both in solution (Ca) and formulation (Fa), as described by Rangel et al. (2020) and Silva Scarpin et al. (2021). The addition of the avobenzone derivative and ethylhexyl methoxycrylene to the formulations (Fb and Fc, respectively) improved the photostability of the UV filters.

It is important to emphasize the correlation between the photodegradation/photoinstability of a compound and its potential harmful effects on biological systems. The use of photounstable molecules can compromise the safety and efficacy of the formulations. These toxicity effects can manifest through the formation of reactive intermediates and degradation products, or even through the degradation of active molecules and modifications to their chemical structure (Gaspar and Maia Campos, 2006). Therefore, it is recommended to perform photoreactivity and phototoxicity tests (ICH, 1996; Damiani et al., 2007; Silva Scarpin et al., 2021; Teixeira, et al., 2021).

Photosensitizing compounds can become toxic when exposed to UV radiation. The first step in this process is the absorption of photons that excite the chromophore. This excitation promotes an electron from the ground state to the higher energy singlet excited state. This molecule in the singlet excited state can either return to its ground state or reach a lower energy triplet excited state, which can trigger photochemical reactions leading to the formation of photoproducts (Shaath, 2010) and the generation of ROS. These ROS reactions can involve the superoxide anion (SA) through type I reaction, or singlet oxygen (SO) through type II reaction, which are the main intermediate species in the phototoxic response. Therefore, the photoreactivity test was based on the generation of ROS, SO and SA, by avobenzone derivative after UVA irradiation (OECD, 2019b).

The avobenzone derivative, formed by the groups A1 (avobenzone), carbonyl connected to a phenol in the *para* position, and a phenol group in the R1 (resveratrol) hydroxyl linked to the aromatic group in the *para* position, was more photostable to UVA radiation than avobenzone itself. It was also determined to be non-photoreactive, precisely due to absence of keto-enol group present in avobenzone, which is responsible to the formation of photoproducts (in this case SO), as discussed by Schwack and Rudolph, (1995), Lhiaubet-Vallet et al. (2010), Shaath (2010) and Freitas et al. (2015).

Due to the photoinstability of avobenzone and octyl methoxycinnamate, it is likely that their photodegradation products or increased ROS levels are responsible for their phototoxicity in the prediction model, damaging the fibroblasts and decreasing cell viability after UVA irradiation (OECD TG 432) (Benevenuto et al., 2015; Kawakami and Gaspar, 2015; Silva Scarpin et al., 2021). The avobenzone derivative, on the other hand, was considered non-phototoxic, which can be correlated to its photostability. When the avobenzone derivative was added to the combination of the photounstable UV filters, it was able to photo stabilize them and consequently decrease their phototoxicity potential. The phototoxicity assay is considered a stand-alone test for negative results due to its high sensitivity, being, generally, the only test required in cases of acute toxicity, when the compound under study is not predicted as phototoxic (Liebsch et al., 2005). It is also the first step of the biological assessment for photosafety prediction, considering that photoreactive compounds, when exposed to UV light, may generate photo irritation, photoallergy and photogenotoxicity reactions (OECD, 2019a).

In the evaluation of the antioxidant activity of the avobenzone derivative, it is important to consider that oxidative stress is a complex process. As a result, complementary methodologies are needed to assess the antioxidant potential of the compounds of interest, taking into account different species of ROS (Alam et al., 2013). For the initial screening of antioxidant activity, *in chemico* methods such as DPPH free radical scavenging are commonly used. This assay is simple, sensitive and low-cost (Chen et al., 2013; Lópes-Alarcón and Denicola, 2013). In this specific methodology, the avobenzone derivative exhibited promising antioxidant potential, with statistically similar IC_50_ values to the antioxidant control, quercetin, a molecule well-known for its antioxidant activity (Song, 2020).

To complement the evaluation of avobenzone derivative’s antioxidant potential, in addition to analytical assays, *in vitro* cell based methodologies were used. This is because a single chemical method does not take into account important biological parameters, such as lipophilicity and bioavailability. Therefore, the ability of compounds to exhibit antioxidant activity in cell culture should also be assessed, as the involvement of different cellular components is essential during oxidative stress (Lópes-Alarcón and Denicola, 2013). The assays involved UVA exposure, since UVA radiation is relevant for ERO production (Zastrow et al., 2009; Vandersee et al., 2015).

In this study, the UVA protective effect of avobenzone derivative was assessed by detecting intracellular ROS immediately after UVA radiation using the probe DCFH_2_-DA, firstly in keratinocytes HaCaT and after in a RHS model. This probe is commonly used to evaluate intracellular ROS, such as hydroxyl radical, hydrogen peroxide, nitrite and carbonate anion. It undergoes hydrolysis by cellular esterases and, in the presence of these ROS, DCFH is converted into its fluorescent derivative DCF, which can be measured using various fluorescence-based techniques (Kalyanaraman et al., 2012). The results showed that the avobenzone derivative, tested at 125, 250, and 500 μg/mL, was able to protect HaCaT cells against ROS generation. However, at the highest tested concentration (1,000 μg/mL) it exhibited a potential pro-oxidant effect. Similar pro-oxidant effects have been reported in the literature for other antioxidant compounds, such as tocopherols, polyphenols and nitric oxide, which may exhibit pro-oxidant properties at concentrations above a certain threshold (Veskoukis, et al., 2019; Bacchetti, et al., 2020).

As mentioned earlier, the avobenzone derivative consists of carbonyl connected to a phenol in the *para* position (A1) and a phenol group in the R1 (resveratrol) hydroxyl linked to the aromatic group in the *para* position, and in between them, an N-acylhydrazone subunit, facilitating conjugation extension. According to observations made by Spiegel et al., (2020), the antioxidant potential of a compound can be enhanced, depending on the position of phenoxyl groups (hydroxyl groups attached to the aromatic ring). These groups play a critical role in the delocalization process, which stabilizes the molecule in the presence of ROS. The authors observed that when two or more phenoxyl groups are in *ortho* or *para* position (as seen in the avobenzone derivative), the molecule has greater stabilization and higher antioxidant activity. There are also reports in the literature suggesting the amine and amide groups (which are present in the avobenzone derivative) are associated with the action of glutathione peroxidase, an enzyme that protects bio membranes and cellular components from oxidative stress. As a result, these groups contribute to the antioxidant potential of this molecule (Bhabak and Mugesh, 2009; Mamgain et al., 2022).

Since the RHS model mimics the biological functions of the skin, with a differentiated epidermis, with stratification and a living dermis (Roguet et al., 1994; Afaq et al., 2009; Lee et al., 2017), it serves as a valuable tool in assessing the antioxidant potential of the avobenzone derivative through the detection of UVA-induced intracellular ROS production. The results showed that the avobenzone derivative was able to protect the RHS model, resulting in a 30.7% reduction of ROS production, confirming its antioxidant potential in monolayer cells. When combined with the photounstable combination of UV filters, avobenzone and octyl methoxycinnamate, the avobenzone derivative reduced ROS production by 39.5%, further confirming its photo stabilizing effect and ability to minimize ROS generation in tissues.

Various methods, including the use of natural and synthetic matrices, have been employed to develop artificial skin models to replace animal testing. These skin models serve as valuable tools for understanding the functional mechanisms of the skin and for conducting transdermal drug and chemical testing (Kumamoto, et al., 2018; Yun, et al., 2018; Hausmann, et al., 2019; Jordão et al., 2024). The RHS model used in this study is particularly important for assessing UV damage, being positioned between monolayer cell cultures, animal models and clinical studies. In this case, we have investigated the effects of a single dosage of UVA radiation accompanied by a single 1 h of treatment with antioxidant compounds. However, it is important to note that repeated exposures and longer treatment durations would provide a more realistic representation of *in vivo* conditions and real-life situations (Lelièvre et al., 2024).

## 5 Conclusion

Overall, the avobenzone derivative was evidenced to possess a potential as a promising ingredient for sunscreens, as it exhibits UV booster properties. It effectively photostabilizes the combination of photounstable UV filters, avobenzone and octyl methoxycinnamate, both in solution and in formulation, reducing their phototoxic potential. Additionally, it primarily also absorbs UVB (280–320 nm) and UVA II (320–340 nm) radiation, as well as a portion of the UVA I (340–360 nm) radiation. Furthermore, it exhibits significant antioxidant potential, both *in chemico* and *in vitro*, protecting against DPPH free radical formation and UVA-induced intracellular ROS formation in HaCaT keratinocytes and a RHS model at concentrations ranging from 125 to 500 μg/mL. While the methodologies employed in this study have certain limitations, the use of skin cells and the RHS model adds relevance to the evaluation of photoprotective potential of novel UV filter derivatives. These findings offer significant insights for the development of new topical materials aiming to protect the skin against UV damage. Although acknowledging the current limitations, further validation and testing could enhance the applicability of these methodologies in real-life situations.

## Data Availability

The original contributions presented in the study are included in the article/Supplementary material, further inquiries can be directed to the corresponding author.

## Abbreviations

BASF, Sunscreen Simulator program; DCF, 2,7-dichlorodihydrofluorescein; DCFH, dichlorofluorescein; DCFH_2_-DA, 2,7-dichlorodihydrofluorescein-diacetate; DMSO, dimethyl sulfoxide; DPPH, 1,1-diphenyl-2-picrylhydrazyl; FDA, Food and Drug Administration; MPE, mean photo effect; NBT, nitroblue tetrazolium; NRU, neutral red uptake assay; NT + UV, untreated irradiated control; NT -UV, untreated non-irradiated control; OECD, Organization for Economic Co-operation and Development; PBS, phosphate-buffered saline; RHS, reconstructed human skin; RNO, reactive nitrogen species; ROS, reactive oxygen species; SA, superoxide anion; SDS, sodium dodecyl sulfate; SO, oxygen singlet; SPF, Sun Protection Factor; TG, test guideline; UVA, ultraviolet ray A (320–400 nm); UVB, ultraviolet ray B (280–320 nm).
